# Cortical ischaemic patterns in term partial-prolonged hypoxic-ischaemic injury—the inter-arterial watershed demonstrated through atrophy, ulegyria and signal change on delayed MRI scans in children with cerebral palsy

**DOI:** 10.1186/s13244-020-00857-8

**Published:** 2020-03-30

**Authors:** Anith Chacko, Savvas Andronikou, Ali Mian, Fabrício Guimarães Gonçalves, Schadie Vedajallam, Ngoc Jade Thai

**Affiliations:** 1 Clinical Research & Imaging Centre, Bristol Medical School, Faculty of Health Sciences, University of Bristol, Bristol, UK; 2 Department of Radiology, Division of Neuroradiology, Children’s Hospital of Philadelphia, Philadelphia, USA

**Keywords:** Cortical ischaemic patterns, Watershed zone, Hypoxic-ischaemic injury, Partial-prolonged hypoxia, Watershed continuum

## Abstract

The inter-arterial watershed zone in neonates is a geographic area without discernible anatomic boundaries and difficult to demarcate and usually not featured in atlases. Schematics currently used to depict the areas are not based on any prior anatomic mapping, compared to adults.

Magnetic resonance imaging (MRI) of neonates in the acute to subacute phase with suspected hypoxic-ischaemic injury (HII) can demonstrate signal abnormality and restricted diffusion in the cortical and subcortical parenchyma of the watershed regions.

In the chronic stage of partial-prolonged hypoxic-ischaemic injury, atrophy and ulegyria can make the watershed zone more conspicuous as a region. Our aim is to use images extracted from a sizable medicolegal database (approximately 2000 cases), of delayed MRI scans in children with cerebral palsy, to demonstrate the watershed region.

To achieve this, we have selected cases diagnosed on imaging as having sustained a term pattern of partial-prolonged HII affecting the hemispheric cortex, based on the presence of bilateral, symmetric atrophy with ulegyria. From these, we have identified those patients demonstrating injury along the whole watershed continuum as well as those demonstrating selective anterior or posterior watershed predominant injury for demonstration.

Recognition of this zone is essential for diagnosing partial-prolonged hypoxic-ischaemic injury sustained in term neonates. The images presented in this pictorial review provide a template for identifying the cortical watershed distribution when there is milder regional (anterior, parasagittal, peri-Sylvian and posterior) watershed injury and for more severe injury where multiple regions are injured in combination or as a continuum.

## Key points

- Lack of clear anatomic structural demarcation of the inter-arterial watershed makes appreciation difficult in normal brains.
- Recognition of this zone and its potential involvement is essential for diagnosing partial-prolonged hypoxic-ischaemic injury in term neonates.
- Watershed injury at any stage should be distinguished from arterial territory involvement.
- In the chronic phase, watershed injury must be distinguished from generalised atrophy of the brain.
- This pictorial review provides a template for identifying the cortical watershed distribution in milder regional or wider more severe injury in a continuum.

## Introduction and aim

Hypoxic-ischaemic injury is a potentially devastating occurrence with significant morbidity and mortality worldwide. Outcomes can be very poor with prognosis dependent on timing, severity and duration of the insult. The patterns of injury are also dependent on maturity of the brain at the time, in addition to the timing, severity and duration of the insult [1–3]. Imaging findings can differ according to gestational age (pre-term [< 36 weeks] vs term infants) as well as being dependent on timing of the investigation [4]. Clinical outcomes can be varied depending on treatment and severity of initial injury, with potentially severe neurologic sequelae and outcomes.

Hypoxia can occur both in utero (inadequate placental perfusion and gaseous exchange due to either foetal or maternal factors) or perinatally/postnatally (e.g. severe hyaline membrane disease, meconium aspiration syndrome, pneumonia or congenital heart disease) [5]. The watershed zone, when affected by hypoxic-ischaemic injury, preferentially demonstrates ulegyria in the areas of atrophy which does not occur in other postnatal causes of this type of injury and ulegyria specifically does not occur in injuries to the brain occurring after the age of 3 months [6].

Brain ischaemia (hypoperfusion) together with hypoxia causes an inflammatory cascade with acidosis, release of inflammatory mediators and free radical formation. These substances lead to loss of normal autoregulation and subsequent diffuse neuronal cell death. In term infants, myelinated fibres are more metabolically active and are therefore more vulnerable to acute hypoxia [1, 7].

Treatment of suspected hypoxic-ischaemic injury is mainly supportive with maintenance of adequate ventilation, vitals and metabolic status. Reported trials have indicated improvement in neurological outcome through a neuroprotective effect in infants with moderate abnormalities who are treated with selective head cooling. Early diagnosis and timely intervention are, therefore, critical in the management [2, 5, 8–10].

The neuroprotective effect of hypothermic therapy in neonates with hypoxic-ischaemic injury may be due to reduction of the secondary energy failure, which is strongly associated with the pattern and severity of injury within the cortex [11–13]. Magnetic resonance imaging performed after hypothermic therapy has shown a reduction in changes related to the basal ganglia, thalami and within the cortical white matter. Studies have also shown no increase in lesions associated with therapeutic hypothermia. Post-cooling findings on conventional sequences can be varied, with up to complete resolution of abnormalities noted in some neonates treated for moderate hypoxic-ischaemic injury [12].

Clinical outcomes and prognosis of hypoxic injury are multifactorial being dependent on the severity of the initial insult, patterns of involvement of cortex and deep grey nuclei, findings on diffusion-weighted imaging (DWI) and magnetic resonance spectroscopy (MRS) as well as electroencephalogram (EEG) [14]. Early MRI changes due to hypoxic-ischaemic injury are most obvious on imaging performed between weeks 1 and 2 post-delivery; however, earlier scanning can make the diagnosis or assist in clinical management [14]. Only subtle abnormalities may be visualised on conventional sequences within the first few days. DWI can identify infarction of the white matter but is not as reliable at detecting significant injury to the basal nuclei and thalami. The appearances of infarction are usually obvious very early and last for approximately 1 week on DWI, after which conventional sequences become more obviously abnormal and assist with diagnosis in the setting of pseudo-normalisation of the DWI sequence [15, 16].

Knowledge of the evolution of acquired lesions or hypoxic injury during the perinatal period is important as there is a predictable evolution of the injuries—serial imaging may therefore assist in the assessment of the timing of injury. This may also be useful when other non-static causes (e.g. metabolic disorders) are being considered [14].

Thorough knowledge of normal development of the brain and of the range of potential acquired lesions in the perinatal period together with their potential evolution is required for accurate interpretation of imaging performed for diagnosis or evaluation in these patients. Perinatal injury can often be symmetric and may appear normal, while normal neonatal brains may appear pathologic to inexperienced radiologists [14].

The long-term neurodevelopmental outcome is dependent on the severity of the injury post hypoxic ischaemia and the resultant encephalopathy. Adverse outcomes are rare in children with mild injury, more common in moderate injury, and almost always present in severe injury [1, 17]. Prognosis can include major or catastrophic outcomes such as severe cognitive impairment, cerebral palsy, or even death. Relatively minor outcomes can include intellectual impairments, difficulties in executive functions, behaviour, and social incompetence. The MRI pattern of injury has been utilised to predict outcomes, with the injury in the watershed being associated with cognitive impairment, often without functional motor deficits [18–20].

**Fig. 1:**
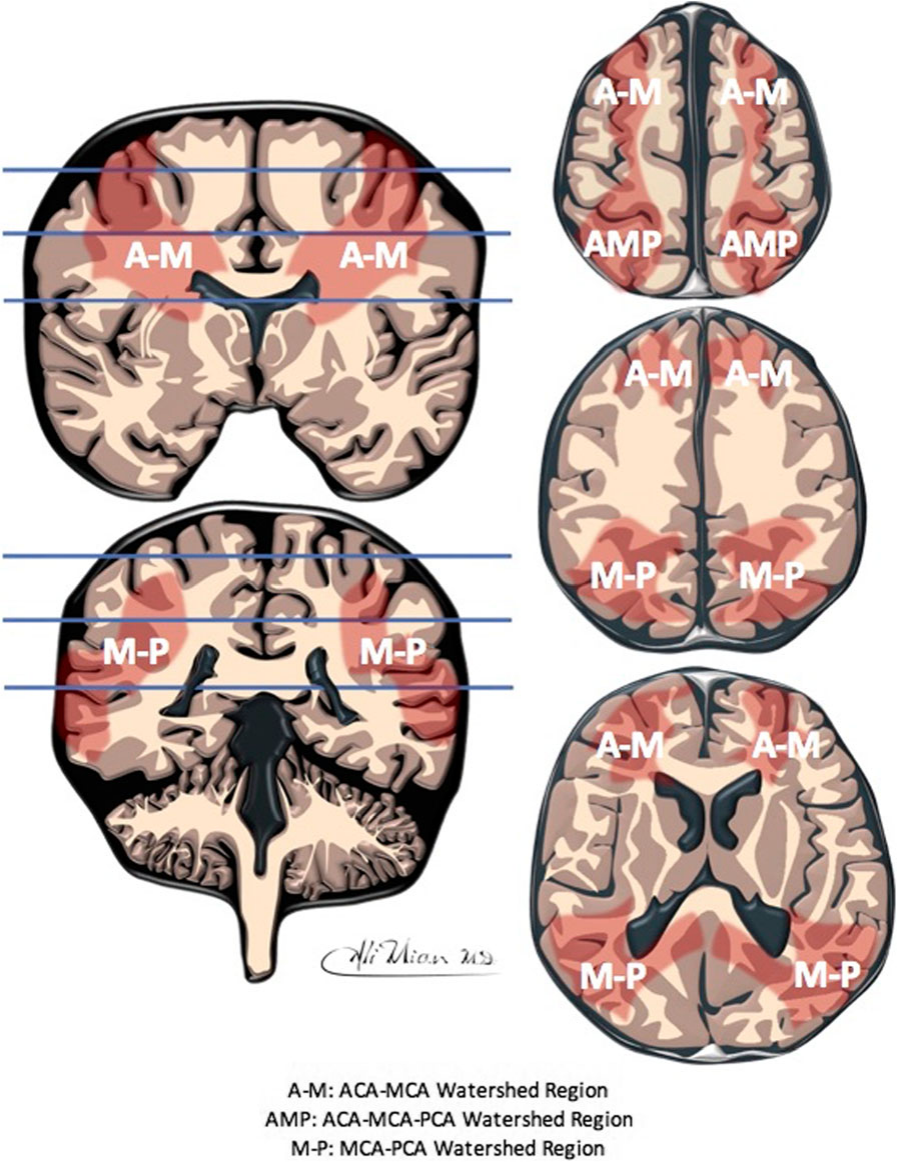
Schematic representation of the inter-arterial watershed regions (depicted in red overlay). The horizontal lines on the coronal images (on the left) depict the levels at which the axial images (on the right) are derived from. The letter annotations depict the confluence between anterior cerebral artery (A = ACA) and middle cerebral artery (M = MCA); ACA, MCA and posterior cerebral artery (P = PCA); and between MCA and PCA

The inter-arterial watershed zone of the brain, also known as the inter-territorial border zone [21], is the portion of the brain that lies at the periphery of the supply of the main vessels of the brain, i.e. between the supply of the anterior cerebral artery (ACA), the posterior cerebral artery (PCA) and the middle cerebral artery (MCA). Since this is a geographic zone without discernible anatomic boundaries, it is not possible to demarcate it directly in normal subjects, and it is therefore not featured in anatomic atlases, especially in neonates. Schematics used for demonstration of the watershed zone in children are not based on any prior anatomic mapping of the region (Fig. 1). In contrast, the watershed zone for adults has been outlined by mapping arterial infarctions from many patients [21].

The watershed zone could be mapped in new-borns with partial-prolonged hypoxic-ischaemic injury, because this region has the poorest supply from the cerebral vessels [22, 23]. The full extent of the watershed is not always involved, however, and multiple cases are required to show the variety of patterns of involvement. The relevance of defining the topography of the watershed, in its full extent and regarding components that can be involved individually, becomes apparent during litigation in children who sustained hypoxic-ischaemic injury in the perinatal period at term gestation. In such scenarios, proving a diagnosis of partial-prolonged hypoxic-ischaemic injury through demonstration of watershed involvement on diagnostic imaging by the plaintiff strengthens the advocacy for long-term support for severely disabled children and their families [6, 24].

Magnetic resonance imaging (MRI) of neonates with suspected hypoxic-ischaemic injury in the acute/subacute phase demonstrates signal abnormality and restricted diffusion in the cortical and subcortical parenchyma of the watershed regions. These signal changes may be challenging to identify on T2-weighted or fluid-attenuated inversion recovery (FLAIR) images and may be even harder to demarcate when they are only visible on diffusion-weighted images (DWI) [4, 6, 23, 25–27]. In contrast, Shroff et al. have observed that in the chronic stage of evolution of partial-prolonged hypoxic-ischaemic injury, atrophy and ulegyria make watershed more conspicuous as a region [4].

Our aim is to use images extracted from a sizable medicolegal database (approximately 2000 cases), of delayed MRI scans in children with cerebral palsy, to demonstrate the watershed region. To achieve this, we have selected cases diagnosed on imaging as having sustained a term pattern of partial-prolonged hypoxic-ischaemic injury affecting the hemispheric cortex, based on the presence of bilateral, symmetric atrophy with ulegyria. From these, we have identified those patients demonstrating injury along the whole watershed continuum as well as those demonstrating selective anterior or posterior watershed predominant injury for demonstration.

The cortical watershed region spans from anterior (between the supply of the anterior and middle cerebral arteries) over the vertex to the posterior (between anterior, middle and posterior cerebral arteries) parts of the cerebral hemispheres on either side (Fig. 1), and therefore cannot be viewed in continuity on standard planar cross sectional imaging. On standard axial cross sectional images, for example, the anterior watershed injury may appear as bilateral triangular wedges of injury separate from posterior watershed injury, despite continuity over the vertex [6, 28]. It is for this reason that the watershed region in children should also be demonstrated on reconstructions designed to demonstrate bilateral symmetric disease involving the cortical surface. We have used multi-planar MRI scans, 3D reconstructions and ‘flat-earth’ maps derived from curved reconstructions [28], to depict typical individual areas of watershed involvement as well as continuity of the watershed zone.

## Delayed features of hypoxic-ischaemic injury affecting the watershed on MRI

Partial-prolonged perinatal hypoxia results in preferential shunting of blood to vital brain structures (primal structures which are essential to survival), such as the brainstem, cerebellum, thalami, basal ganglia, and hippocampi, at the expense of the cerebral cortex and white matter of the inter-vascular boundary (watershed) zones [3]. The result of this reduction of blood flow to the cortex is cortical thinning and reduction of the underlying white matter volume in the watershed zones, as the injury evolves [29, 30].

**Fig. 2:**
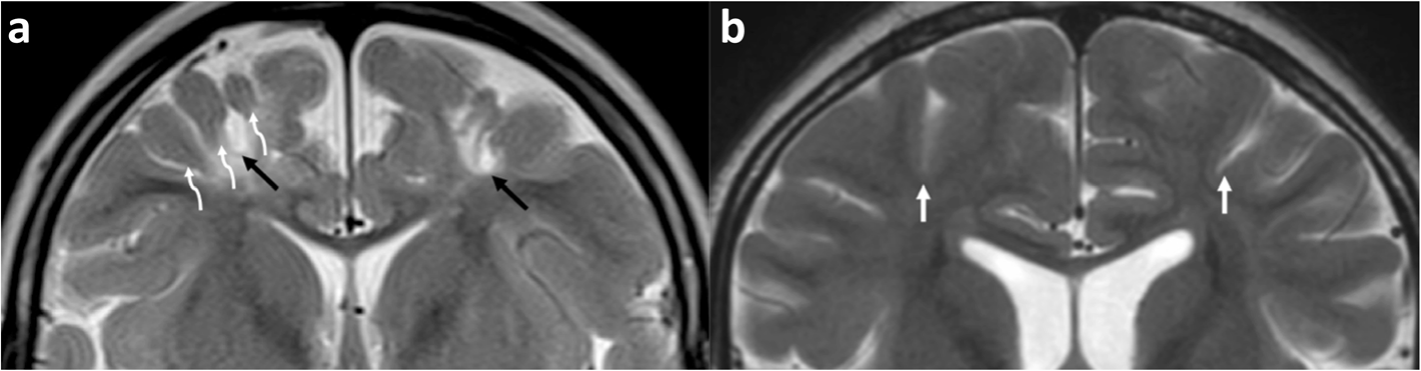
**a**, **b** Ulegyria in a patient with partial-prolonged HII in prolonged labour (**a**) compared with normal gyral and sulcal pattern (**b**). Coronal T2-weighted image in **a**, a 4-year-old boy, who suffered a partial-prolonged HII demonstrating bilateral, symmetric localised atrophy in the parasagittal regions and abnormal underlying white matter high signal. There is ulegyria with overt thinning of the stems (curved white arrows) of the involved gyri and a resultant teardrop shape to the sulcus (black arrows). In comparison, the gyri in **b**, a normal 8-year-old girl, have parallel walls throughout their length and the sulci remain slit like from their surface to their depth (straight white arrows)

In the clinical setting of imaging older children with cerebral palsy resulting from prior perinatal partial-prolonged HII (as opposed to acute-phase imaging in new-borns with hypoxic-ischaemic encephalopathy), it is crucial to be able to distinguish regional atrophy of the watershed zones from generalised atrophy (which can be caused by a number of diverse conditions including dehydration, malnutrition, end-stage metabolic disorders, severe epilepsy, post-viral infection, in utero conditions, and HIV) [6, 24, 29]. Additionally, the regional watershed atrophy from hypoxic-ischaemic injury can have the appearance of ulegyria [4, 6, 31–34]. This is a pattern involving the gyri in the chronic phase where there is thinning of the bases (stems) of the gyri with resultant focal enlargement of the sulci at their depth, and relative sparing of the crests of the gyri [6, 35]. These atrophied gyral ‘stems’ are considered a ‘watershed within the watershed’ because they are more susceptible to HII than the crests of the gyri [6, 36, 37]. Inexperienced or general radiologists can also appreciate ulegyria by looking for the ‘tear-drop’ shape of the widened sulcus at its depth, rather than look for thinning of the gyral stems (Fig. 2).

### Distribution patterns of watershed involvement in HII

**Fig. 3:**
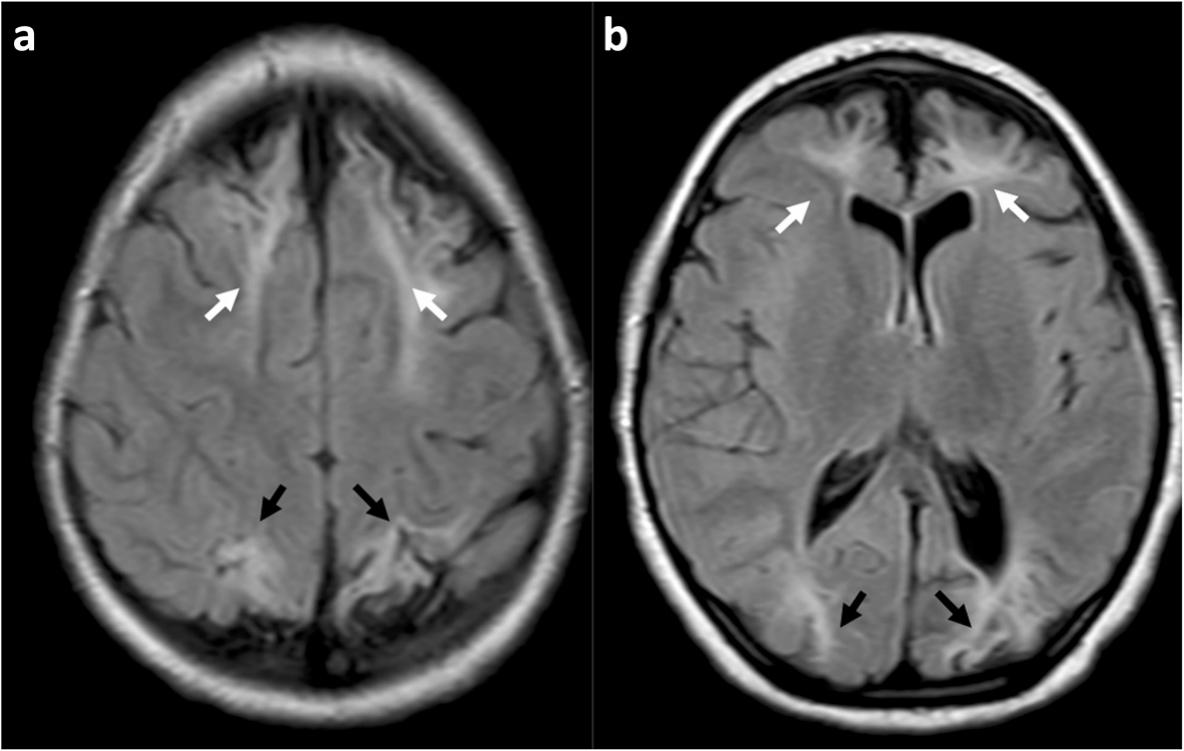
**a**, **b** Sequential axial FLAIR images in a 4-year and 9-month-old girl, who sustained a partial-prolonged hypoxic-ischaemic injury at term gestation during prolonged labour, demonstrating bilateral, symmetric volume loss with abnormal underlying white matter high signal in the anterior watershed (white arrows), apparently discontinuous from similar involvement of the posterior watershed (black arrows). This is perceived as a classical watershed distribution but does not demonstrate the watershed continuum in its entirety

Descriptions of the watershed region in texts often include images showing anterior and posterior triangles of the brain between arterial territories on an axial cross sectional image [38]. This is considered a classic presentation (Fig. 3) but fails to demonstrate that the watershed spans a continuous zone from the anterior hemispheres, over the vertex to the posterior hemispheres.

Indeed, the whole watershed is not always involved as a continuous area when there is a global hypoxic-ischaemic injury. Some regions are more often involved than others, depending on individual susceptibility and severity of the insult.

#### Peri-Sylvian and posterior inter-vascular watershed (PIVWS)

**Fig. 4:**
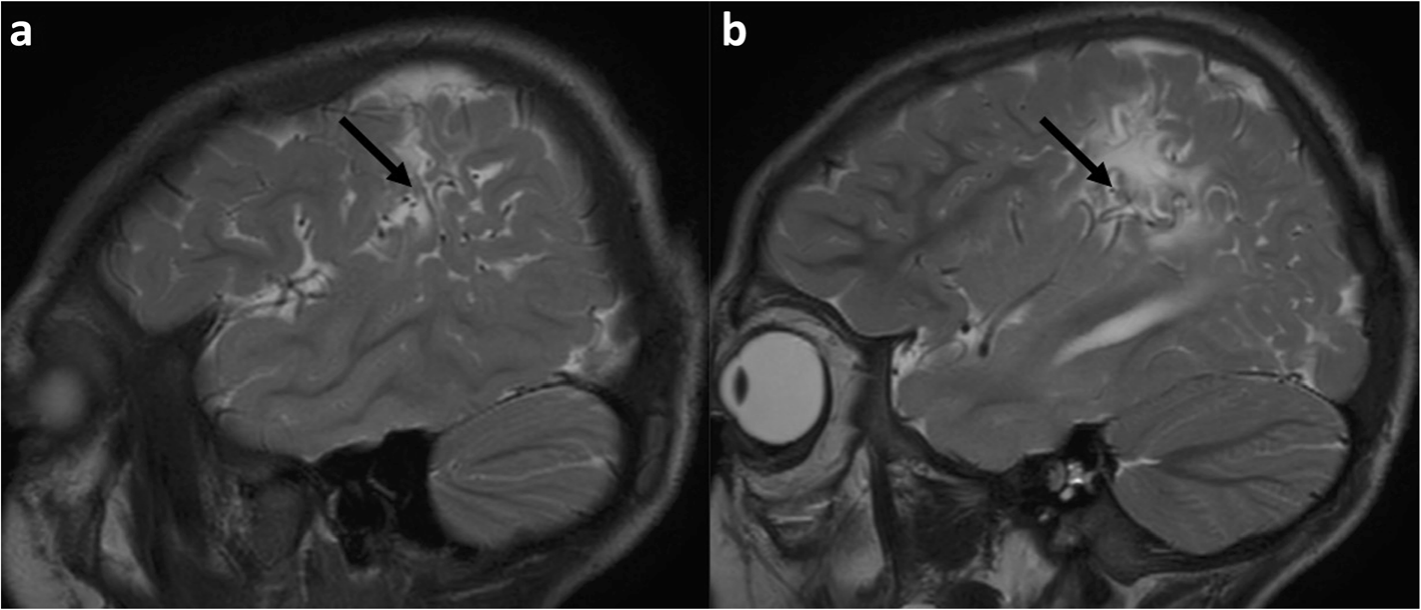
**a**, **b** Isolated peri-Sylvian watershed involvement in a 12-year-old girl. Sequential T2-weighted MRI slices in the sagittal plane demonstrate atrophy of the cortex and subcortical signal abnormality with white matter volume loss, at the most superior portion of the Sylvian fissure (black arrows), which represents an ‘end-zone’ of all three major cerebral arteries and represents the most commonly involved portion of the watershed zone in patients sustaining partial-prolonged HII at term gestation

**Fig. 5:**
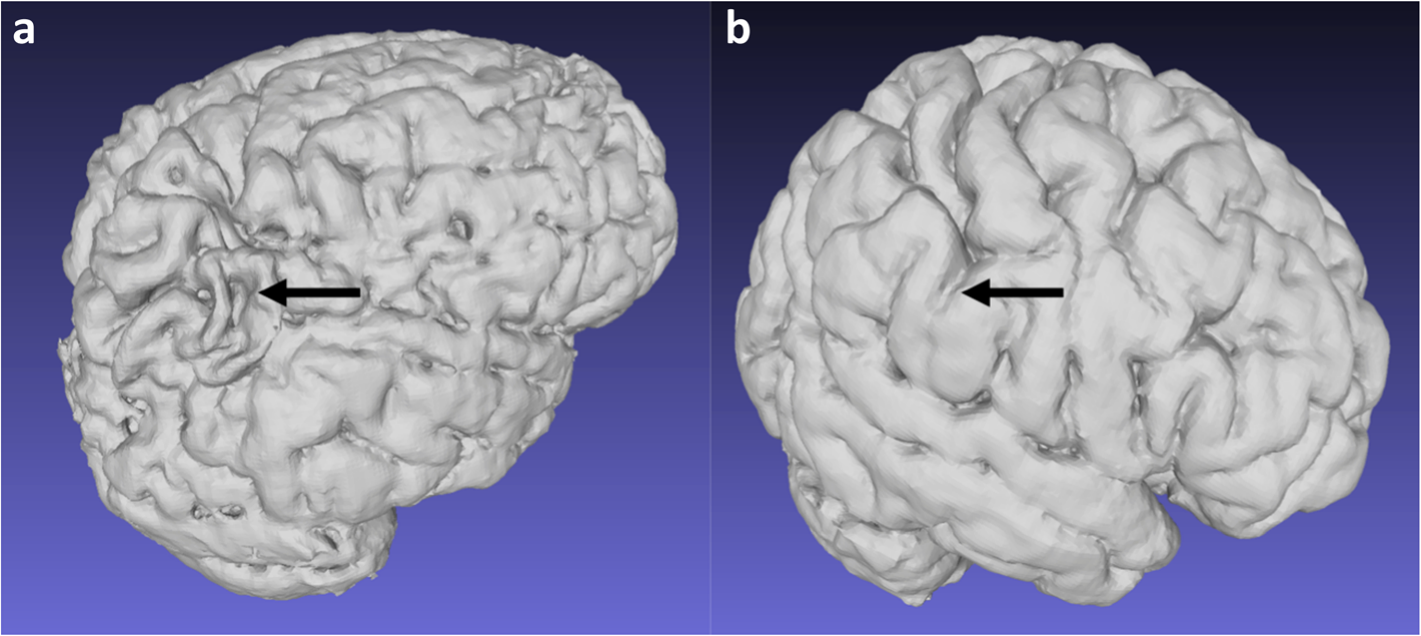
**a**, **b** 3D reconstructions of the brain after skull stripping and segmentation of the cortex. Isolated peri-Sylvian involvement in a 10-year-old girl (**a**) compared with a normal 12-year-old girl (**b**) serving as a control. The volumes demonstrate isolated atrophy in the peri-Sylvian regions (black arrow) in the child with prior partial-prolonged HII (**a**) as compared to the normal thickness of the gyri (black arrow) in the control (**b**)

It is reported that the parieto-occipital and posterior temporal lobes are typically more affected than the anterior watershed [29]. This predilection represents the region most susceptible to hypotension and a fall in perfusion because it represents the ‘end-field’ between all three main cerebral arteries [39]. This region is more often referred to as ‘peri-Sylvian’ involvement and often merges with the posterior inter-vascular watershed dorsally and with the parasagittal watershed anteriorly. Isolated peri-Sylvian involvement is best appreciated on sagittal images at the far lateral aspects of the cerebral hemispheres (Figs. 4 and 5).

**Fig. 6:**
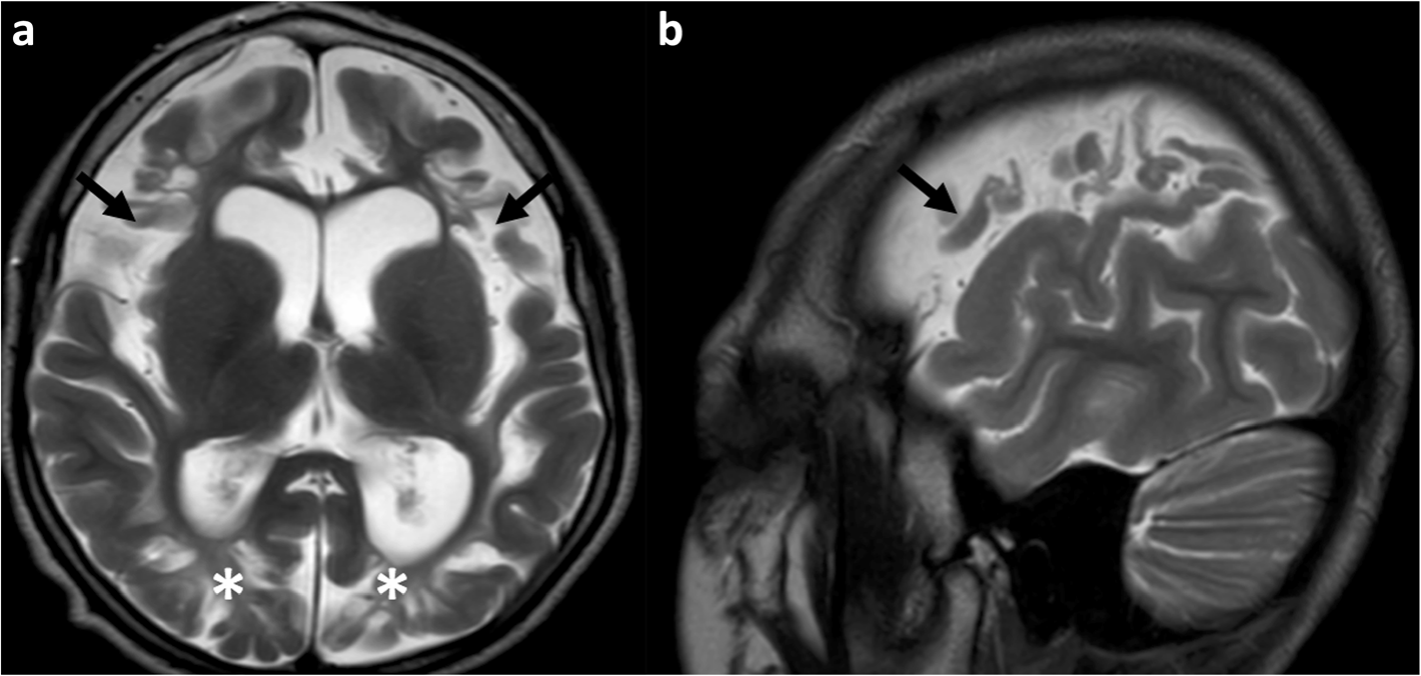
**a**, **b** Axial (**a**) and sagittal (**b**) T2-weighted MRI in a 6-year-old boy who sustained a partial-prolonged hypoxic-ischaemic injury at term gestation, demonstrates extension of injury from the para-falcine portion of the parasagittal zones, inferiorly anteriorly along the Sylvian fissure (involving the peri-Sylvian zone), with involvement of the inferior aspect of the frontal lobe (arrows) and sparing of the superior temporal lobe. There is also ulegyria in the posterior inter-arterial watershed region (asterisks), visible on the axial T2 image (**a**)

Anterior extension of involvement from the peri-Sylvian watershed (which lies between all three major arterial territories at the posterior aspect of the Sylvian fissure) is commonly along the Sylvian fissure, but more often affects the frontal lobe side, with relative sparing of temporal lobe side of the parenchyma around the Sylvian fissure (Fig. 6).

**Fig. 7:**
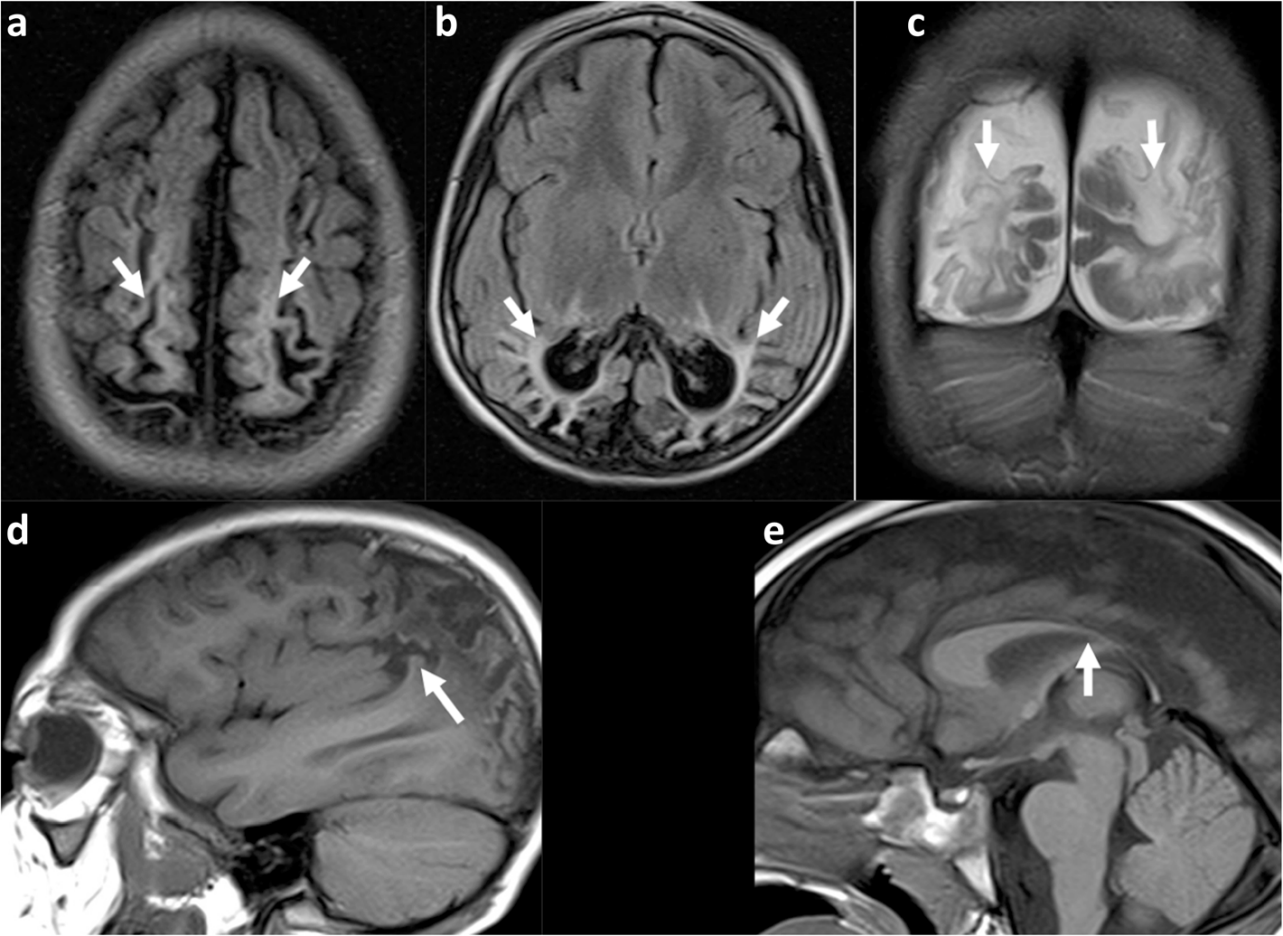
**a**–**e** Peri-Sylvian and posterior watershed injury in a 4-year-8-month boy. **a**, **b** Axial FLAIR images at two different levels which not only demonstrate the peri-Sylvian and posterior watershed distribution of the atrophy (arrows) but also demonstrate the underlying white matter signal abnormality up to the ventricular edge, with white matter volume loss. **c** Coronal T2-weighted imaging confirms the atrophy and signal abnormality of the posterior watershed in another plane (arrows) and demonstrates relative sparing of the occipital lobes just above the tentorium. **d** Sagittal T1-weighted image demonstrates the peri-Sylvian distribution of atrophy (arrow). **e** Sagittal midline T1 shows the marked volume loss of the posterior body, isthmus, and splenium of the corpus callosum (arrow) corresponding to cortical and white matter volume loss in the posterior watershed

**Fig. 8:**
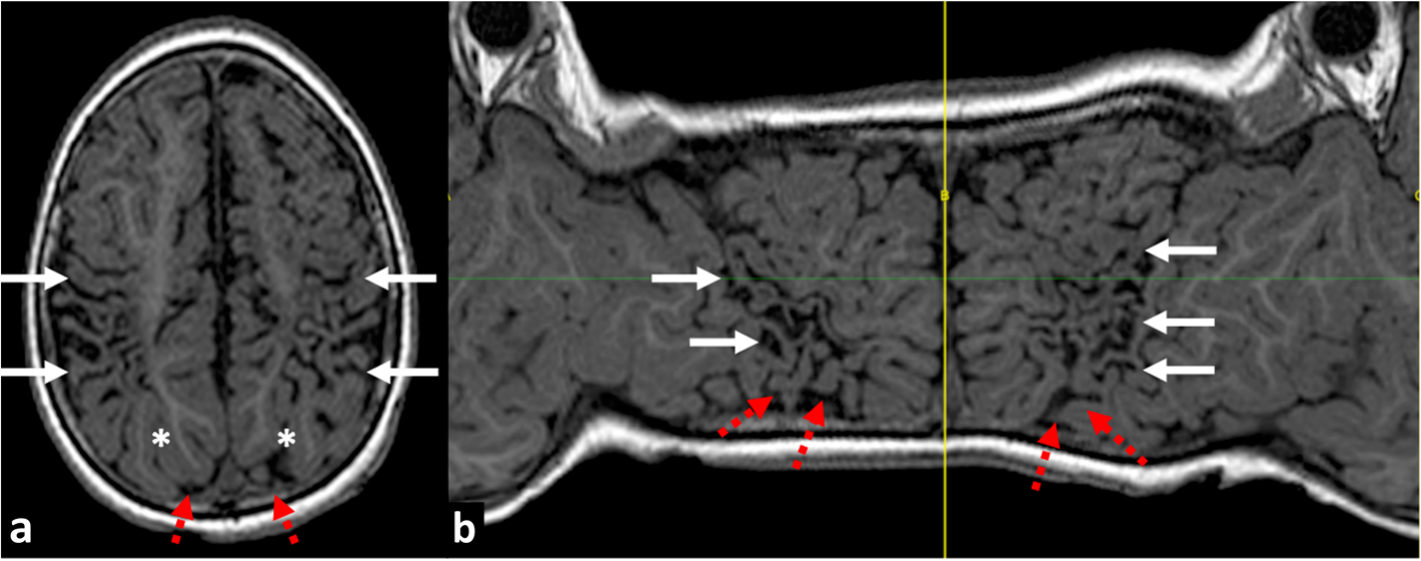
**a** Axial reference T1W image and (**b**) flat earth Mercator map derived from curved reconstructions of 3D T1-weighted images in a 6-year-old girl demonstrating atrophy and ulegyria in the peri-Sylvian (white arrows) and posterior watershed (red dashed arrows) distribution in a child who suffered partial-prolonged hypoxic-ischaemic injury perinatally at term gestation. Note sparing of the anterior and parasagittal watershed regions as well as the temporal lobe portions of the peri-Sylvian watershed. Also, note the asterisk depicting the apparent discontinuity on the axial image which is clearly shown to be continuous on the Mercator map

**Fig. 9:**
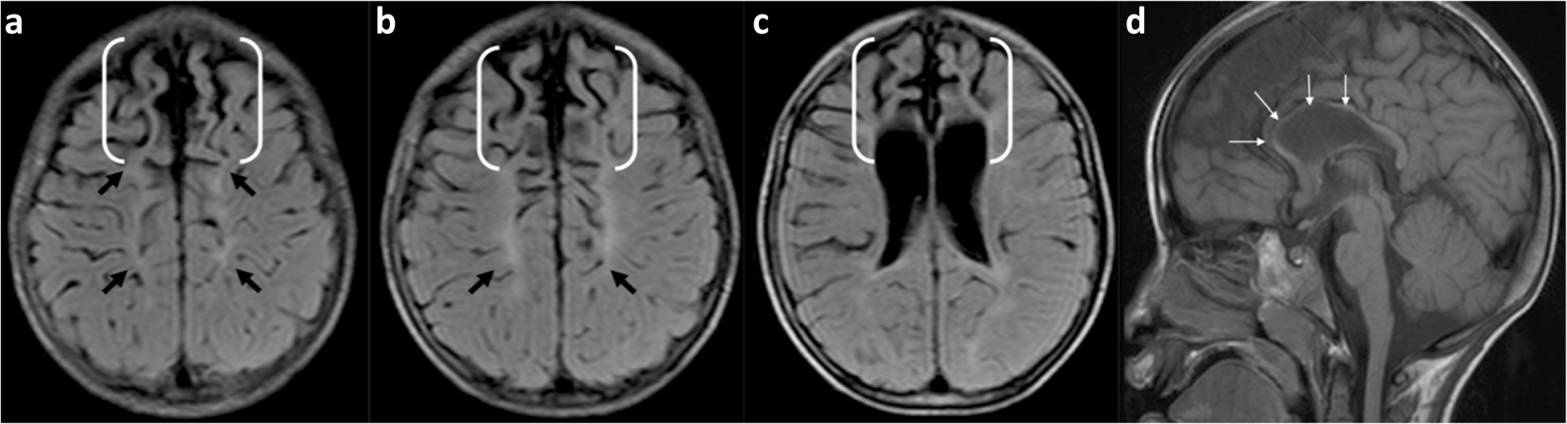
**a**–**d** An 8-year and 10-month-old boy on sequential axial FLAIR images from superior to inferior (**a**–**c**), demonstrating the bilateral, symmetric, predominantly anterior inter-vascular watershed involvement, manifesting as frontal atrophy and underlying signal abnormality in continuity with the parasagittal volume loss and signal abnormality (black arrows). There is an expansion of the inter-hemispheric fissure anteriorly and prominent, deep, superior frontal sulci bilaterally (white brackets). The sagittal T1-weighted midline image (**d**) demonstrates associated corpus callosum volume loss predominantly affecting the rostrum, genu, and anterior body (thin arrows)

Extension to involve the posterior inter-vascular watershed is best seen on axial and coronal images and often spares the calcarine cortex and the occipital lobes just above the tentorium, unless there is severe disease or if there is associated perinatal hypoglycaemia, which affects the occipital lobes and thalamic pulvinars [40]. Corpus callosum thickness is known to reflect the volume of white matter [41, 42] and regional corpus callosum thinning of its posterior components, including the splenium, almost invariably accompanies posterior watershed volume loss (Figs. 7, 8, and 9).

#### Parasagittal and anterior inter-vascular watershed (AIVWS)

Parasagittal cerebral injury is a specific pattern of cerebral lesion that is considered characteristic of milder forms of hypoxic-ischaemic encephalopathy affecting full-term new-borns [6, 34]. This distribution refers to the location of injury involving the cerebral convexities on either side of the midline inter-hemispheric fissure and superomedial hemispheres [13]. This includes cortical necrosis and necrosis in the immediately subjacent white matter bilaterally [6]. More severe prolonged hypoxic-ischaemic injury can result in extension into a greater part of the lateral cerebral convexity [34].

**Fig. 10:**
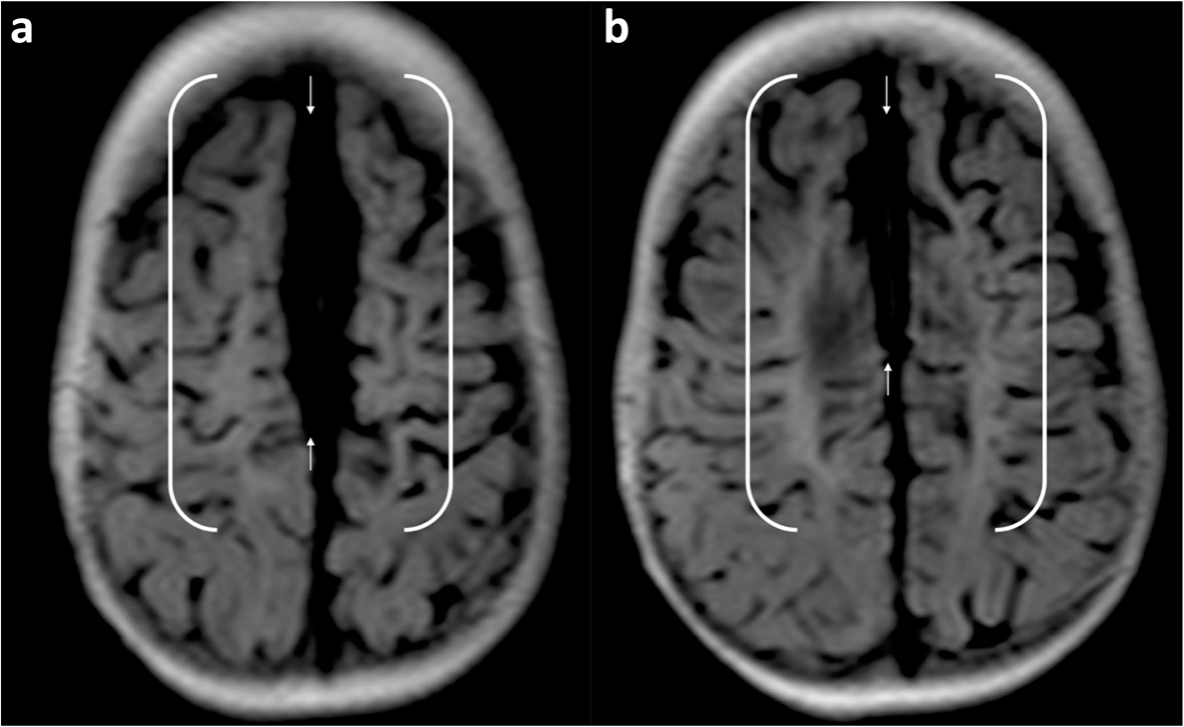
**a**, **b** Sequential axial FLAIR images in a 2-year-old girl who sustained a partial-prolonged HII at term gestation, demonstrating volume loss and abnormal signal involving the parasagittal and anterior inter-vascular watershed regions in continuity (between white brackets) and sparing of the peri-Sylvian and posterior watershed. There is bi-convex expansion of the inter-hemispheric fissure at the anterior vertex (white arrows) indicating the predominant parasagittal involvement

**Fig. 11:**
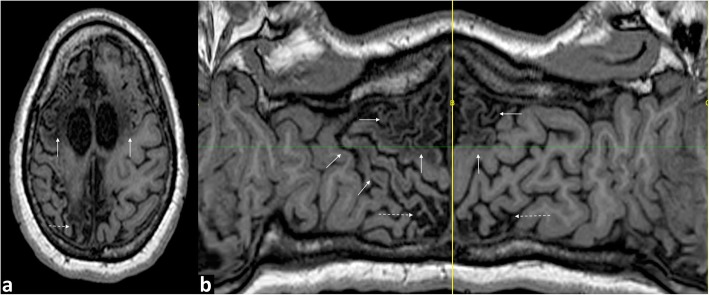
**a** Axial T1W image and (**b**) flat earth Mercator map derived from a curved reconstruction of a 3D T1 sequence in a 16-year-old girl who sustained a partial-prolonged HII at term gestation, demonstrating predominantly anterior watershed and parasagittal volume loss with cystic encephalomalacia, within this involved area, in continuity (solid white arrows) as well as posterior watershed volume loss (dashed white arrows)

**Fig. 12:**
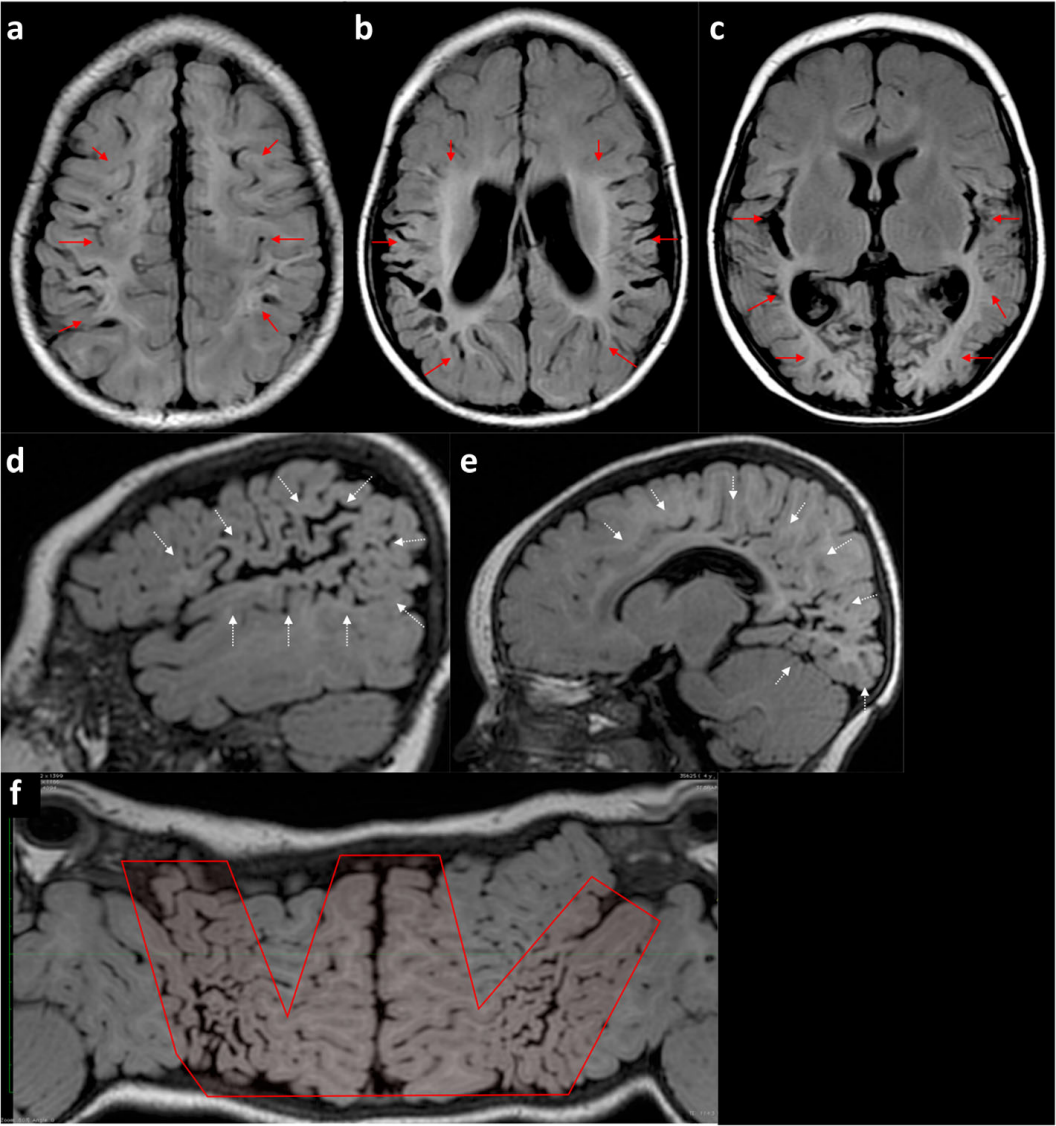
**a**–**f** A 1-year-old boy who sustained a previous partial-prolonged hypoxic-ischaemic injury at term gestation. Sequential axial FLAIR images (**a**–**c**) from the vertex to the level of the lateral ventricles demonstrate volume loss with ulegyria and underlying white matter abnormal high signal affecting the watershed continuum in order from anterior, through parasagittal and peri-Sylvian to posterior (solid red arrows). **d**, **e** Sequential sagittal FLAIR images from lateral to medial demonstrate the same volume loss and underlying abnormal signal involving the parasagittal, peri-Sylvian and posterior watershed as a continuum (dotted white arrows). **f** Mercator map overview of the cerebral surface created from a curved reconstruction of 3DT1 images confirms atrophy of the watershed as a continuum from anterior, through parasagittal and peri-Sylvian to posterior (inside schematic ‘W’)

On MRI, parasagittal injury is seen on either side of the falx (para-falcine) with resultant expansion of the inter-hemispheric fissure, which can be bi-convex when localised, and as enlargement of the superior frontal sulci with surrounding signal abnormality. Superior frontal sulcus expansion manifests as deep projections of the widened sulci into the cerebral hemispheres. Anterior watershed injury is often noted in continuity with parasagittal involvement and manifests as bi-frontal atrophy and ulegyria with subjacent white matter abnormal signal extending antero-inferiorly. Concomitant thinning of the genu of the corpus callosum and expansion of the anterior body of the lateral ventricle corresponds to volume loss in these regions (Figs. 10, 11, and 12).

#### The complete watershed distribution—the ‘Watershed Continuum’

**Fig. 13:**
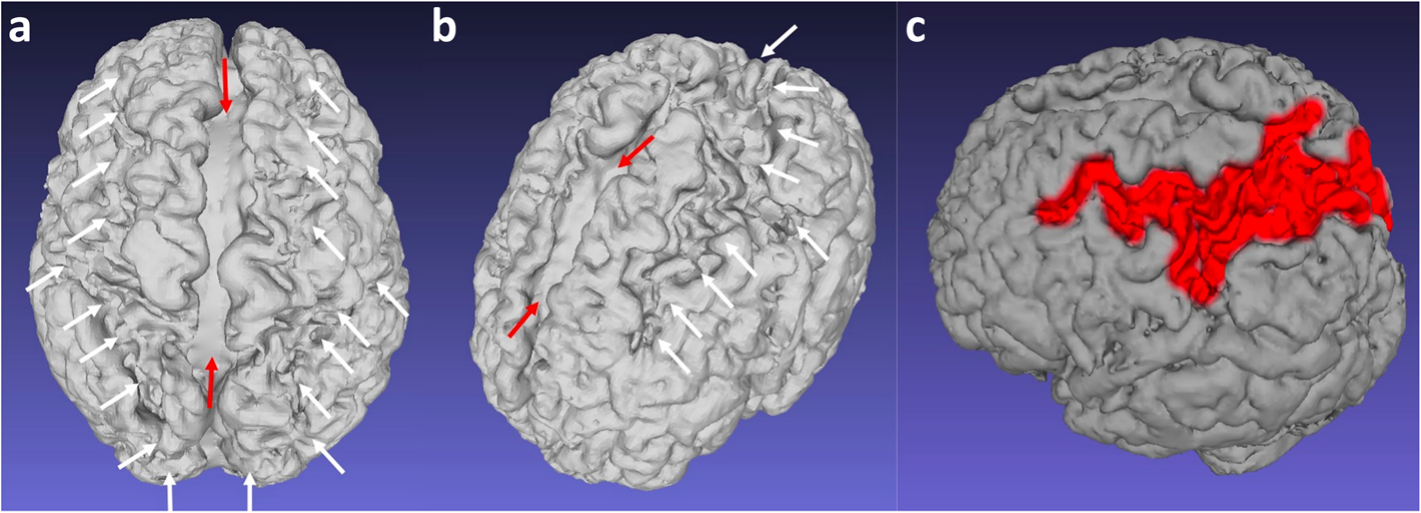
**a**–**c** 3D reconstruction of the cerebral surface after skull stripping and segmentation in a 2-year-old boy who sustained a partial-prolonged hypoxic-ischaemic injury at term gestation. **a** View of the vertex from above from posterior and (**b**) view of the left hemisphere from above anterior demonstrate continuity of atrophy involving the anterior, parasagittal, peri-Sylvian and posterior inter-arterial watershed zones (white arrows). The inter-hemispheric fissure shows localised bi-convex expansion at the regions worst affected (brackets). **c** Lateral oblique with the affected posterior inter-vascular watershed region depicted in red—note the continuity with the para-falcine watershed anteriorly and posteriorly

**Fig. 14:**
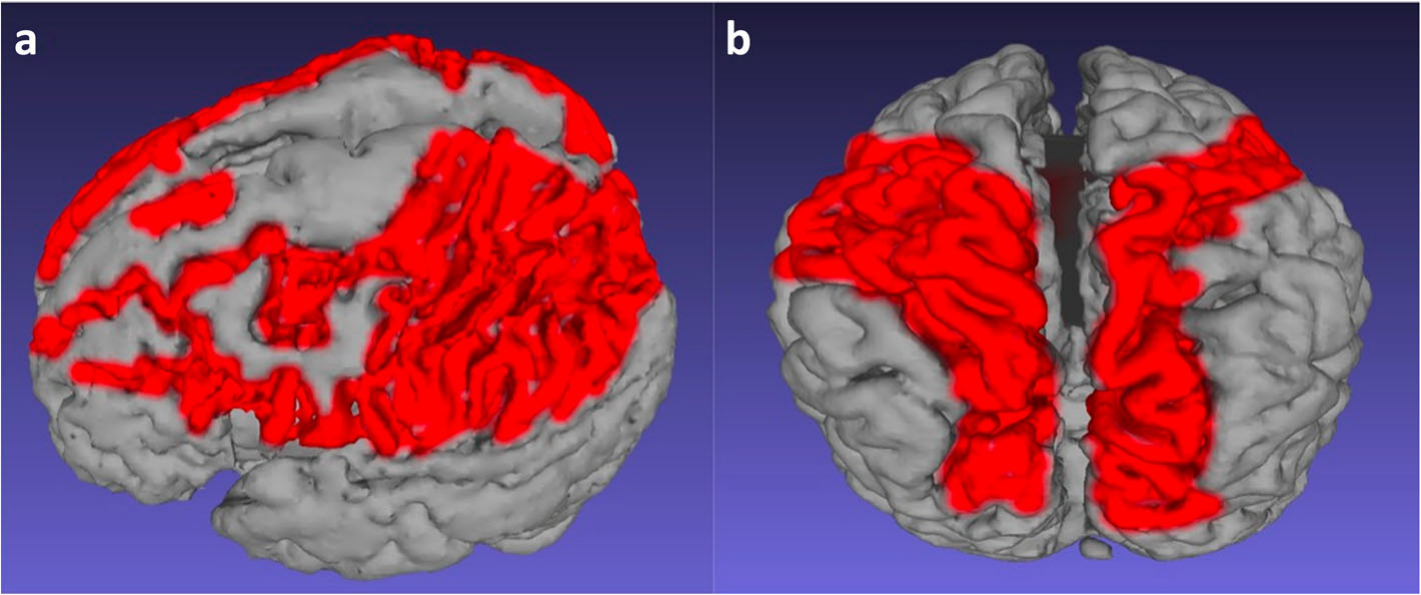
**a**, **b** 3D reconstructions of the brain after skull stripping and segmentation in 2 different children who sustained a partial-prolonged hypoxic-ischaemic injury at term gestation, demonstrating atrophic cortical surfaces in variations of the watershed continuum (painted in red). **a** Left side view of a 10-year-old girl. **b** Vertex view from above of a 2-year-old boy

Partial-prolonged hypoxic-ischaemic watershed injury is generally bilateral, involving an area including the anterior lobes and posterior convexity and with lesions usually quite symmetric [43]. There is no definitive cut-off between the anterior watershed and the parasagittal watershed, between the parasagittal and peri-Sylvian watershed, or between the peri-Sylvian and posterior watershed. Therefore, the watershed should be considered as a continuum that can be involved in totality or in part, depending on the patients’ inherent susceptibility and severity of the injury. 3D models and flat earth reconstructions in extensive watershed injury provide a means to appreciate the shape of the watershed continuum (Figs. 12, 13, and 14).

#### Extensive/severe disease

**Fig. 15:**
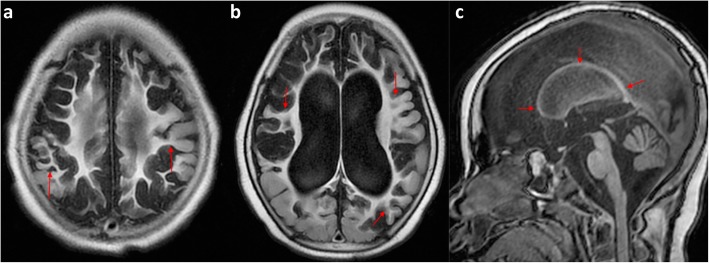
**a**–**c** MRI imaging in a 5-year-old girl who sustained a severe partial-prolonged hypoxic-ischaemic injury at term gestation. The sequential FLAIR images from the vertex (**a**) to the level of the lateral ventricle bodies (**b**) demonstrates severe volume loss of both cortex and subcortical white matter extending to involve the deep white matter of the entire cerebral hemisphere bilaterally, with only a strip of periventricular white matter remaining and showing abnormal high signal throughout. There is also prominent ulegyria (arrows) of the watershed continuum from anterior, over the vertex, through the peri-Sylvian region, and into the posterior watershed. The sagittal midline T1-weighted image (**c**) demonstrates corresponding corpus callosum thinning throughout its length (arrows)

**Fig. 16:**
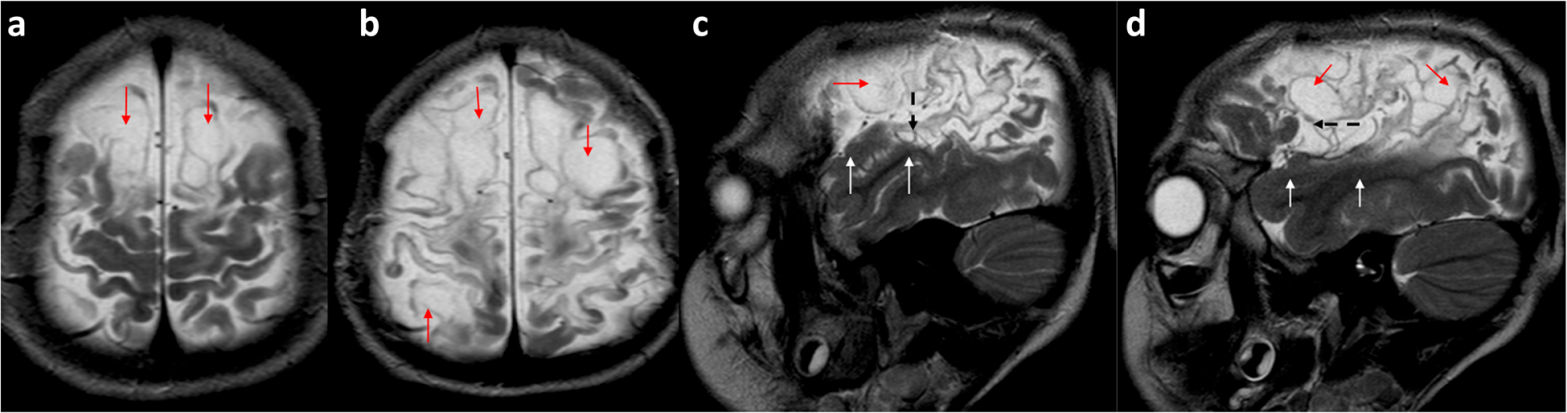
**a**–**d** T2-weighted imaging of a 6-year-old boy who sustained a severe/catastrophic prolonged hypoxic-ischaemic injury at term gestation. Axial image just below the vertex (**a**) and sagittal image of the right lateral peri-sylvian region (**b**) shows extensive injury of the watershed continuum represented by multi-cystic encephalomalacia (red arrows). Of note, **c** and **d** are the extension of peri-Sylvian involvement into the posterior temporal lobe and extension of the parasagittal cystic encephalomalacia up to the Sylvian fissure (dashed black arrows) but with preservation of the superior temporal gyrus anteriorly seen on the sagittal images (solid white arrows)

In severe cases, hypoxic-ischaemic damage may extend beyond boundary zones and involve more of the frontal, parietal and temporal lobes (Fig. 15) [3]. In these cases, a watershed predominance should be sought and ulegyria may be a helpful indicator that the cause of the atrophy is hypoxic-ischaemic injury. In the most severe cases, liquefaction of involved areas of cerebral parenchyma results in the formation of multiple cystic areas and this is termed multi-cystic encephalomalacia (Fig. 16).

### Pitfalls

**Fig. 17:**
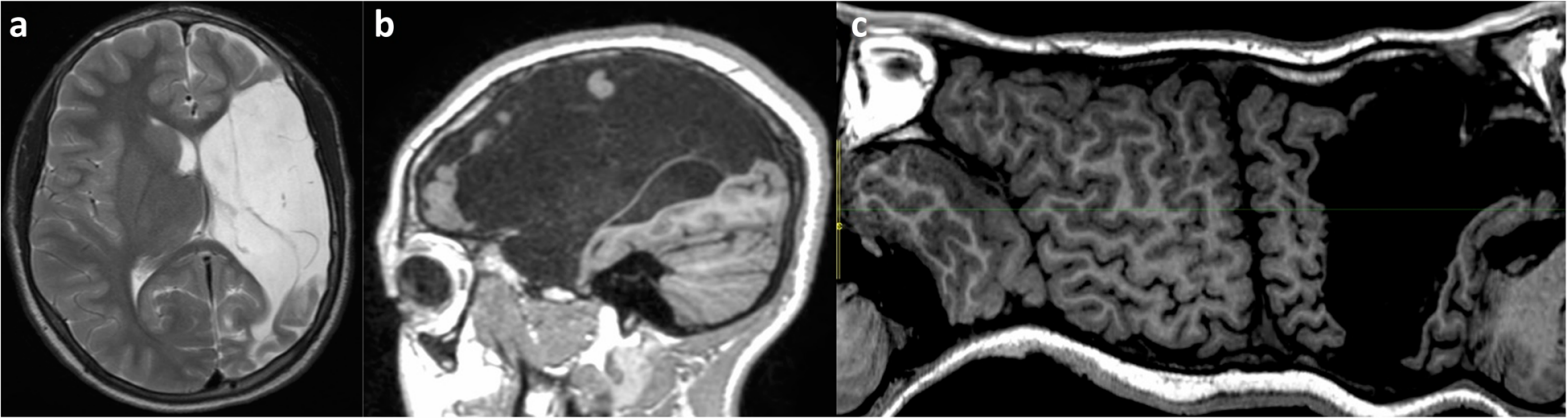
**a**–**c** MRI scan in a 14-year-old girl with a chronic left MCA infarct. Axial T2-weighted image (**a**), sagittal T1-weighted image (**b**) and Mercator map derived from the curved reconstruction of 3D T1 images (**c**) show the unilateral sharply demarcated cystic encephalomalacia involving the arterial territory of the left MCA. Notably, there is involvement of the left temporal lobe, the deep nuclei and the inferolateral aspect of the frontal lobe with sparing of the parasagittal region and superior frontal gyrus, i.e. the watershed. The involvement of the superior temporal lobe which is not a feature of parasagittal extension in severe partial-prolonged hypoxic-ischaemic injury in term neonates can be appreciated, especially on the sagittal image (**b**). The Mercator map derived from the curved reconstruction of 3D T1 images (**c**) confirms the unilateral left MCA territory cystic change and provides an overview comparing the left with the right side of the brain. It also shows that the inter-hemispheric fissure and superior frontal gyrus are relatively preserved (i.e. not expanded/separated through atrophy)

**Fig. 18:**
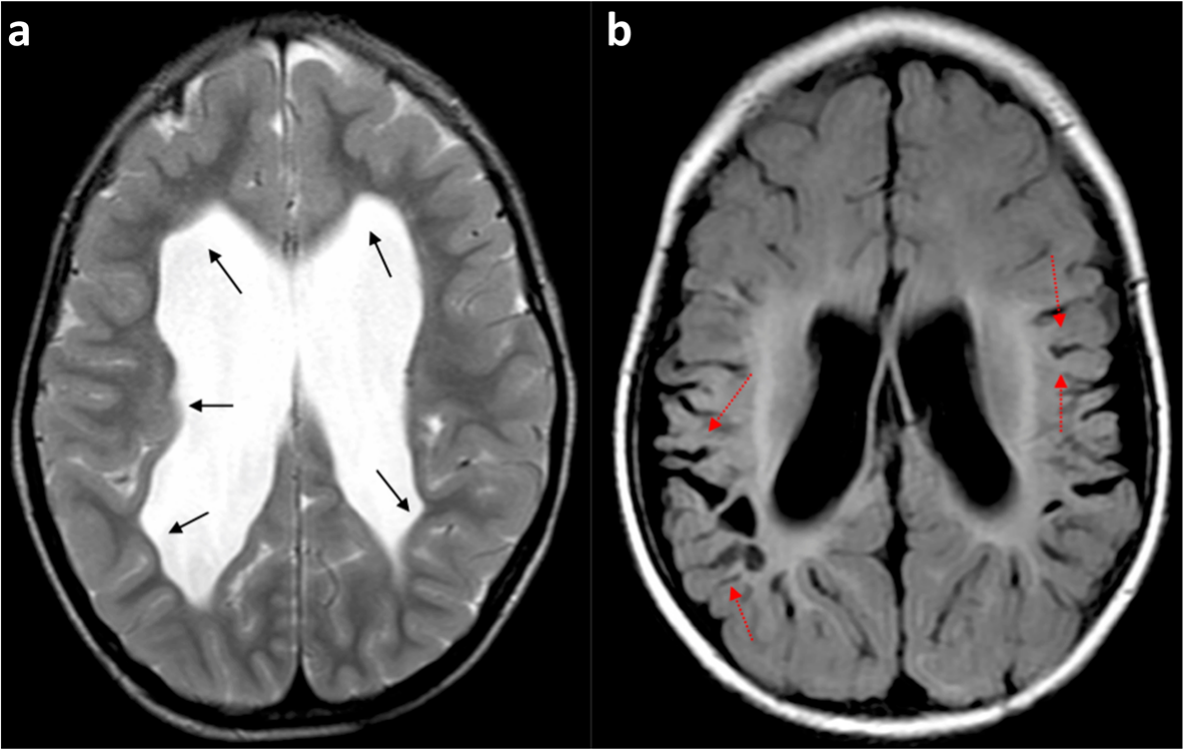
**a**, **b** Comparison of (**a**) Axial T2-weighted image showing periventricular leukomalacia (PVL) with predominantly periventricular white matter volume loss in a 6-year-old boy who sustained a global hypoxic-ischaemic injury as a premature neonate, compared with (**b**) axial FLAIR image showing predominantly subcortical white matter loss in a 1-year-old boy who sustained a severe partial-prolonged hypoxic-ischaemic injury at term gestation. In the patient born prematurely with PVL (**a**), there is such severe periventricular white matter volume loss that the cortical mantle at the sulcal depths abuts the ventricular ependymal edges, causing a ‘wavy’ ventricular margin (black arrows). In addition, the width of the sulci is of equal size at their depth as at their surface, with sulci and gyral surfaces remaining parallel. The patient born at term with partial-prolonged hypoxic-ischaemic injury (**b**) demonstrates preservation of some of the periventricular white matter substance (even though of abnormally high signal) and regular ventricular margins as the cortical mantle at the sulcal depth is separated from the ventricular ependymal edge. There is also ulegyria at the peri-Sylvian regions bilaterally (red dotted arrows)

**Fig. 19:**
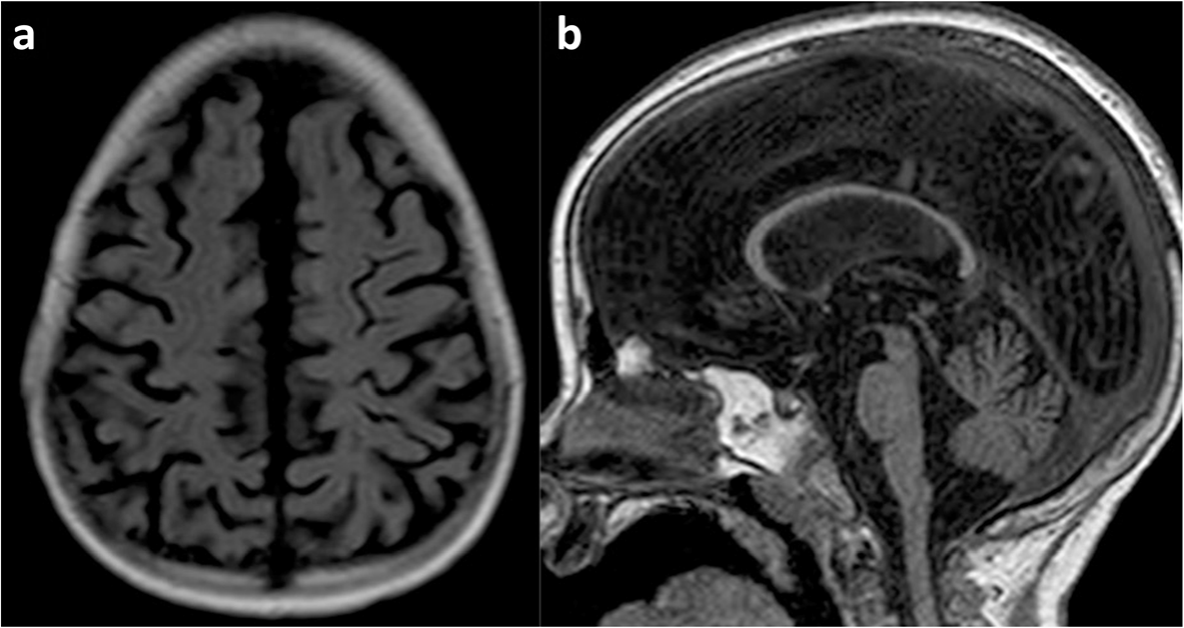
**a**, **b** Generalised atrophy, of unknown cause, in a 5-year-old girl. Axial FLAIR image (**a**) demonstrates generalised atrophy without any regional predominance in the watershed distribution and there is no ulegyria. The midline sagittal T1 image (**b**) demonstrates that there is diffuse moderate thinning of the corpus callosum but also without any regional predominance

Radiologists may find themselves trying to distinguish watershed injury from arterial infarction (Fig. 17), from previous periventricular leukomalacia (Fig. 18) suffered in a premature neonate and from generalised atrophy (Fig. 19) due to any number of other causes.

Bilateral arterial infarction involving both MCA territories is an uncommon finding and should correspond to the MCA supply territory. It regularly involves the temporal lobe, whereas extensive peri-Sylvian and parasagittal disease is usually restricted to the frontal and parietal lobes. This is best appreciated on sagittal imaging.

White matter volume loss from periventricular leukomalacia (PVL) mostly involves the white matter immediately adjacent to the ventricles (Fig. 18). This brings the cortical mantle at the depth of the sulci near the ventricular ependymal lining, causing it to display a scalloped or wavy outline. In contrast, the deep white matter involved in extensive-term watershed HII preserves a layer immediately adjacent to the ventricles (even if this is injured and has an abnormal signal). The ventricular margin in term HII therefore usually remains regular.

Generalised atrophy may be confused with watershed atrophy (Fig. 19) or vice versa. There is however no regional predominance in generalised atrophy and there will be a lack of ulegyria (representing a watershed within the watershed). In generalised atrophy, the width of the sulci at their depth is of equal size to the width of the sulci at their more superficial aspect, indicating that gyral surfaces are parallel.

## Conclusion

The inter-arterial watershed is not clearly defined by specific anatomic boundaries and is therefore difficult to demarcate in a normal brain. However, recognition of this zone is essential for diagnosing partial-prolonged hypoxic-ischaemic injury sustained in term neonates, which also has implications for compensation in litigation. The set of images presented in this pictorial review provides a template for identifying the cortical watershed distribution when there is milder regional (anterior, parasagittal, peri-Sylvian and posterior) watershed injury and for more severe injury where multiple regions are injured in combination or as a continuum using delayed magnetic resonance imaging, with the watershed region represented by regional atrophy, ulegyria and white matter signal abnormalities. This can enable distinguishing between partial-prolonged hypoxic-ischaemic injury and other causes of neonatal encephalopathy.

## Data Availability

The datasets used and/or analysed during the current study and used as a basis for this article are potentially available from the authors on reasonable request.

## Abbreviations

3D: Three dimensional
ACA: Anterior cerebral artery
AIVWS: Anterior inter-vascular watershed
DWI: Diffusion-weighted imaging
EEG: Electroencephalogram
FLAIR: Fluid-attenuated inversion recovery
HII: Hypoxic-ischaemic injury
HIV: Human immunodeficiency virus
MCA: Middle cerebral artery
MRI: Magnetic resonance imaging
MRS: Magnetic resonance spectroscopy
PCA: Posterior cerebral artery
PIVWS: Posterior inter-vascular watershed
PVL: Periventricular leukomalacia
